# Bench to Bedside of Neural Stem Cell in Traumatic Brain Injury

**DOI:** 10.1155/2012/141624

**Published:** 2012-09-17

**Authors:** Solomon O. Ugoya, Jian Tu

**Affiliations:** Australian School of Advanced Medicine, Macquarie University, 2 Technology Place, North Ryde, Sydney, NSW 2109, Australia

## Abstract

Traumatic brain injury (TBI) is one of the leading causes of major disability and death worldwide. Neural stem cells (NSCs) have recently been shown to contribute to the cellular remodelling that occurs following TBI and attention has been drawn to the area of neural stem cell as possible therapy for TBI. The NSCs may play an important role in the treatment of TBI by replacing the damaged cells and eventual remyelination. This paper summarized a critical assessment of recent data and developed a view comprising of six points to possible quality translation of NSCs in TBI.

## 1. Introduction

Traumatic brain injury (TBI) has remained a major cause of mortality, morbidity and leading cause of large-scale disabilities worldwide. TBI results in a large number of deaths and a cause of permanent disabilities with enormous losses to individuals, families, and communities [1]. World Health Organization (WHO), in 2004, has estimated that 25% of road traffic collisions requiring admission to a hospital suffered TBI [1–3].

Moreover, WHO has introduced the new metric tool, the disability-adjusted life year (DALY), which quantifies the burden of diseases, injuries and risk factors. The worldwide leading causes of TBI include road traffic accidents that were estimated being 41.2 million DALYs in 2008, violence being responsible for 21.7 million DALYs, and self-inflicted injuries being 19.6 million DALYs, respectively. All these will leave disability associated with TBI in survivors [2, 3].

However, no effective therapy or program is available for treatment of individuals with TBI; nonetheless, researchers had tried some therapeutic agents like levodopa/carbidopa and some neurotrophic factors in brain injury with persistent vegetative state with the aim of augmenting and slowing the progression from persistent vegetative state into some degree of consciousness. This still needs experimentation to confirm if these dopamine precursors and other neurotrophic factors have any role in TBI. Several other therapeutic agents like cannabinoid dexanabinol, erythropoietin, and gamma-glutamylcysteine ethyl ester have all shown to have neuroprotective effect in human at experimental stage with remarkable improvement in post-TBI outcome [4–8].

Recently, more attention has been drawn to the area of stem cell therapy, largely due to advanced knowledge about stem cells. The stem cells may play an important role in the treatment of TBI by replacing damaged cells, and helping functional recovery. The search for stem cell therapy for TBI is progressing. Since the pathophysiology of TBI is largely unknown, it makes a search for an effective stem cell therapy difficult. This is because multiple cell types like neuronal cells, glial, and endothelial cells are usually involved in TBI. Furthermore, cerebral vasculature, especially the blood brain barrier (BBB), may be affected in TBI; this injury may be focal or diffuse axonal injury (DAI). Taming these burgeoning effects of TBI will require NSCs which can differentiate into neurons and glial cells. It has been reported that progenitor cells differentiated into neurons and glial in adult brain, and an increase in astrocytic progeny is forming reactive astrocytes to primarily limit cyst enlargement in posttraumatic syringomyelia [9–12]. 

This review is an optional extra to see if we can achieve the translation of basic knowledge of neural stem cells into therapeutic options in persons with TBI by enhancing and integrating these neural progenitor cells (NPCs) unto neurogenesis and directing these cells to the specified targets or through multipotency where the transplanted cells can differentiate into glial cells, neurons, and endothelial cells, as the injuries are not always selective but diffuse and we may need to induce these transplanted cells into appropriate phenotype. This is a critical review of existing current literature on neural stem cell research and proposing an approach for quality clinical translation in TBI. We will look at the pathophysiology of TBI and proposing the “six-point schematic approach” to achieve standard and quality bench to bedside in neural stem cell of TBI. We also highlighted the need for suitable clinical translation, coordination, and administration of research in the field of neural stem cell therapy of TBI.

## 2. Pathophysiology of TBI 

 Pathophysiology of TBI involves two main phases: these are primary injury following the trauma, and the secondary injury which is mediated by inflammatory response to trauma. 

### 2.1. Primary Injury

**Figure 1:**
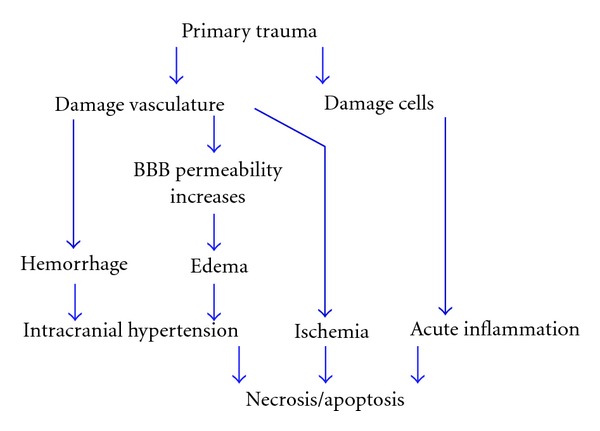
Sequential events of primary injury in TBI. Initial impact is usually by directing trauma to the head either open or closed head injury. This trauma will cause mechanical damage to neurons, axons, glia, and blood vessels by shearing, tearing, or stretching. Blood vessel ruptures cause hemorrhage. Even in unruptured blood vessels, BBB permeability increases resulting in edema. Hemorrhage and edema often lead to intracranial hypertension. Following hemorrhage, ischemia could occur in brain tissue. TBI-caused cell damage induces macrophage and lymphocytes migrant to the injury site releasing inflammatory mediators that triggers a cascade of events towards necrosis and/or apoptosis. Necrosis and/or apoptosis also can be a consequence of hemorrhage and ischemia.

Pathophysiology of initial injury has been postulated to include acceleration, deceleration, and rotational forces which may or may not be as a result of the trauma. This flow of events leads to initiation of inertia which is both acceleration and rotational head movements. This impact on the cortical and subcortical brain structures causes focal or diffuse axonal injury (DAI) and these inertial forces will disrupt the BBB [13]. The primary events also involve massive ionic influx referred to as traumatic depolarization. The major inflammatory neurotransmitters released are excitatory amino acids. This may explain the pathophysiology of DAI in TBI. This is followed by cerebral edema with associated increase in intracranial pressure, which usually forms the major immediate consequences of TBI. Brain edema may come from astrocyte swelling and disruption of the BBB [14, 15]. The BBB is disrupted in acute phase of severe TBI. The expression of high levels of glucose transporter 1 (GLUT 1) was observed in capillaries from acutely injured brain, which occurs in association with compromised BBB function. Vascular endothelial growth factor also plays a role in neuronal tissue disruption and increases the permeability of the BBB via the synthesis and release of nitric oxide [16].  Figure 1 depicts the pathophysiology of the primary injury.

### 2.2. Secondary Injury

**Figure 2:**
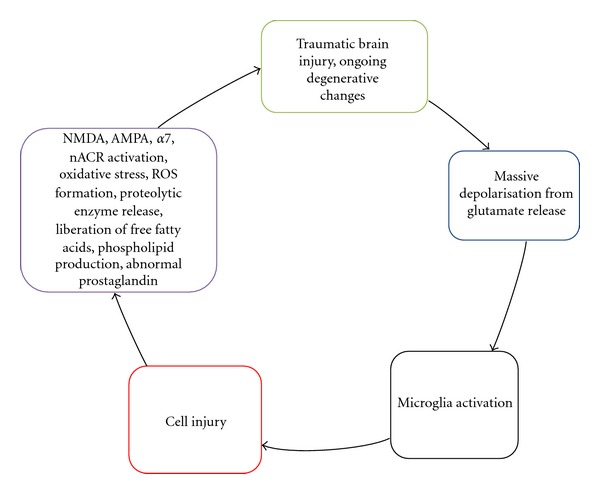
Sequential events of secondary injury in TBI. This includes variety of processes such as depolarization, disruption of ionic homeostasis and release of neurotransmitters, lipid degradation, and oxidative stress. These events are a result of interaction between the excitatory amino acid released with an influx of oxygen-free radicals that ultimately set up NMDA, AMPA, *α*7, and nACR to sustain the unstable environment for cell injury and degenerative changes.

 The secondary events are a complex association of the inflammatory response initiated by the trauma leading to diffuse neuronal degeneration of neurons, glial, axonal tearing, and genetic predisposition (Figure 2). Furthermore, excitatory amino acid release, oxygen radical reactions, and nitric oxide production will lead to activation of N-Methyl-D-aspartate (NMDA), 2-amino-3-(5-methyl-3-oxo-1,2-oxazol-4-yl)propanoic acid (AMPA), alpha-7 nicotinic receptor (*α*7), and nicotinic acetylcholine receptor (nACR) [17–19] and subsequent calcium influx. All these cascades of events will cause mitochondrial disruption and free radical release with eventual tissue peroxidation. One theory is that excitatory amino acid release leads to calcium influx into neurons and other brain cells which promote oxygen-free radical reactions. High calcium and the presence of free-radical molecules create an unstable environment in the cell that may lead to increased production and release of nitric oxide and excitatory amino acids (e.g., glutamate). Nitric oxide may participate in oxygen radical reactions and lipid peroxidation in neighboring cells [20]. A summary is shown in Figure 2. The secondary injury plays a major role in the outcome of TBI. Therapeutic interventions should target this phase as it is the major determinant of morbidity and mortality in TBI [16]. Genes implicated to influence the outcome of TBI include *apoe*. *Apoe* multifactorially affects the clinicopathological consequences of TBI [21]. *Apoe* is associated with increased amyloid deposition, amyloid angiopathy, larger intracranial hematomas, and more severe contusional injury. *Comt* and *drd2* are genes which may influence dopamine-dependent cognitive processes, such as executive or frontal lobe functions. The *ace* gene may affect TBI outcome *via* alteration of cerebral blood flow and/or autoregulation and the *cacna1a* gene may exert an influence *via* the calcium channel pathways and its effect on delayed cerebral edema [22]. Increased signal transducers and activator of transcription (STAT) 3 signaling has been reported in a rat model of TBI [23]. Although several potential genes that may influence the outcomes following TBI have been identified, future investigations are needed to validate these genetic studies and identify new genes that might contribute to the outcomes following TBI.

## 3. Application of NSCs in TBI 

There are at least two possible strategies involving neural stem cells (NSCs) to repair injured brain. They are transplantation of exogenous NSCs and stimulation of endogenous NSCs. 

### 3.1. Transplantation of Exogenous NSCs

There have been attempts to transplant various types of cells, such as neurons and neural stem cells to repair damaged brain. The main objectives of these transplantation experiments are (1) growth facilitation: the transplant fills the lesion site and serves as a cellular bridge; (2) new neurons: the transplant can provide new neurons, which in turn provide new targets and sources of innervations and thus repair the damaged neural circuits; (3) factor secretion: the transplant can produce a variety of substances, such as neurotrophic factors, that may aid in the repair process [24]. Several characteristics of NSCs make them potentially suitable for repair after TBI. Firstly, they can serve as a renewable supply of transplantable cells by clonally expansion in culture. Secondly, they are of CNS origin and the cells generated from the grafts have neural characteristics. Thirdly, NSCs can be manipulated by genetic engineering methods to produce specific proteins, such as neurotrophins, neurotransmitters, and enzymes [25]. 

It has been reported that autologous-cultured cells harvested at time of emergency surgery from patients with TBI and subsequently engrafted into damaged part of the brain can be detected using MRI [26]. The efficacy of transplantation largely depends on a grafting method that optimizes the survival of the transplanted cells and minimizes the graft-induced lesion. Most transplantation studies involved intraparenchymal injection into the CNS, in which cells were grafted directly into or adjacent to the lesion [27–29]. The optimal time for transplantation may not be immediately after injury. The levels of various inflammatory cytokines (TNF*α*, IL-1*α*, IL-1*β*, and IL-6) in the injured brain peak 6–12 hours after injury remain elevated until the 4th day. Although these inflammatory cytokines are known to have both neurotoxic and neurotrophic actions, they are believed to be neurotoxic within a week after injury, which causes the microenvironment to be unsuitable for survival of the grafted cells [30]. However, if too much time passes after the injury, glial scar forms a barrier around the lesion site and inhibits local blood circulation which is needed for graft survival. Thus, it is considered that those 7 to 14 days after injury are the optimal time for transplantation [31, 32].

### 3.2. Stimulation of Endogenous NPCs

Since the description of endogenous neurogenesis in adult brain by Luskin in 1997 [33] and Alvarez-Buylla and co-workers in 2000 [34], several publications have confirmed their findings. They demonstrated the presence of NSCs in adult rodent ventricular zone (VZ) that migrated to the olfactory bulb and integrated into the neuronal network called the rostral migratory stream (RMS). 

However, the potential success of stimulating endogenous NPCs is hinged on delivery of various growth factors. More so, this seems to be the most common way to stimulate NPCs. The following growth factors have been reported: EGF, FGF-2 [35–37], bFGF [38], aFGF [39], BDNF [40], NGF, NT-3 [40, 41], VEGF [42], GDNF [43], IGF-1 [42], and SDF-1 alpha [44]. They were administrated by intraventricular [35], intraparenchymal [40, 42, 45] or intrathecal [36–38, 43] injection. They were reported not only to enhance the proliferation, migration, and gliogenesis of NPCs [35–37, 44] but also to protect the spinal cord from further damage [41, 42]. In addition, these growth factors facilitated the regrowth of axons and remyelination [39, 40, 46]. Functional recovery was also reported after they were delivered into injured spinal cord [35–37, 39]. However, the details of functionary recovery are still not clear. 

Not only growth factors, other molecules, were shown to stimulate endogenous NPCs. Proliferation of endogenous NPCs was demonstrated when the sodium channel blocker tetrodotoxin and the glycoprotein molecule sonic hedgehog were injected into the parenchyma [47, 48]. Imitola and colleagues reported that cognate chemokine receptor type 4 (CXCR4) expressed by NSCs can regulate their proliferation and direct their migration towards the injury site [44]. In addition, antibodies blocking IL-6 receptors were reported to not only inhibit differentiation of endogenous NSCs into astroglia *in vivo *and *in vitro*, but also to promote functionary recovery [49, 50]. Okano and colleagues assumed that the functionary recovery is probably due to blocking IL-6 and consequently inhibiting the formation of glial scars and promoting axonal regeneration [49, 51]. Notably, studies of ATP-binding cassette (ABC) transporters have emerged as a new field of investigation. ABC transporters (especially ABCA2, ABCA3, ABCB1, and ABCG2) are found to play an important role in proliferation and differentiation of NSCs [45, 52–56]. 

In contrast to transplantation of exogenous NPCs, stimulation of endogenous NPCs to repair damaged spinal cord has three main advantages: (1) there is no ethical issue of embryonic and foetal cells, (2) it is usually less invasive, and (3) no immunogenicity; it avoids immunorejection that observed in transplantation of exogenous NPCs [57]. 

Like adult NPCs transplantation studies in SCI, no neurogenesis has been reported from the stimulation of endogenous NPCs. Yamamoto and colleagues reported that lack of neuronal differentiation is related to upregulation of the Notch signal pathways [58]. The increased level of various cytokines within the microenviroment surrounding the area of injury may also cause a lack of trophic support for differentiation into neuronal lineage [59–62]. 

Recently, more attention has been drawn to CBP/p300-phosphorylated Smad complex. It was found that CBP/p300-phosphorylated Smad complex can be bound in NSCs, which may decide the differentiation of NSCs. If the complex is bound with phosphorylated STAT 3, the NSCs differentiate into astroglia lineage cells. On the other hand, if the complex is bound with proneural-type of the basic helix-loop-helix (bHLH) factor, such as neurogenin 1 and 2, they differentiate into the neuronal lineage [51, 63, 64]. Apart from that, Peveny and Placzek reported that *SOX* gene may also play an important role in neural differentiation [65]. 

Once NSCs decide to differentiate into neuronal lineage, a cascade of hundreds of genes is regulated over time to lead the immature neuron into its mature phenotype. Many of these neural genes are controlled by RE1-silencing transcription factor (REST). REST acts as a repressor of neural genes in nonneural cells, while regulation of REST activates large networks of genes required for neural differentiation [66–68]. 

## 4. Bench to Bedside Translation of Stem Cell Therapy

 The main purpose of scientific studies is to put our discoveries into daily clinical practice. The basic science laboratory takes its observations obtained at cellular or molecular levels in a cutting edge condition and implements this into acceptable practice clinically to the benefit of the public. However, this is always met with a lot of challenges, such as ethics, governmental regulations, funding constraints, paucity of adequate collaboration among clinical and basic science, and the challenges of conducting a clinical study.

**Figure 3:**
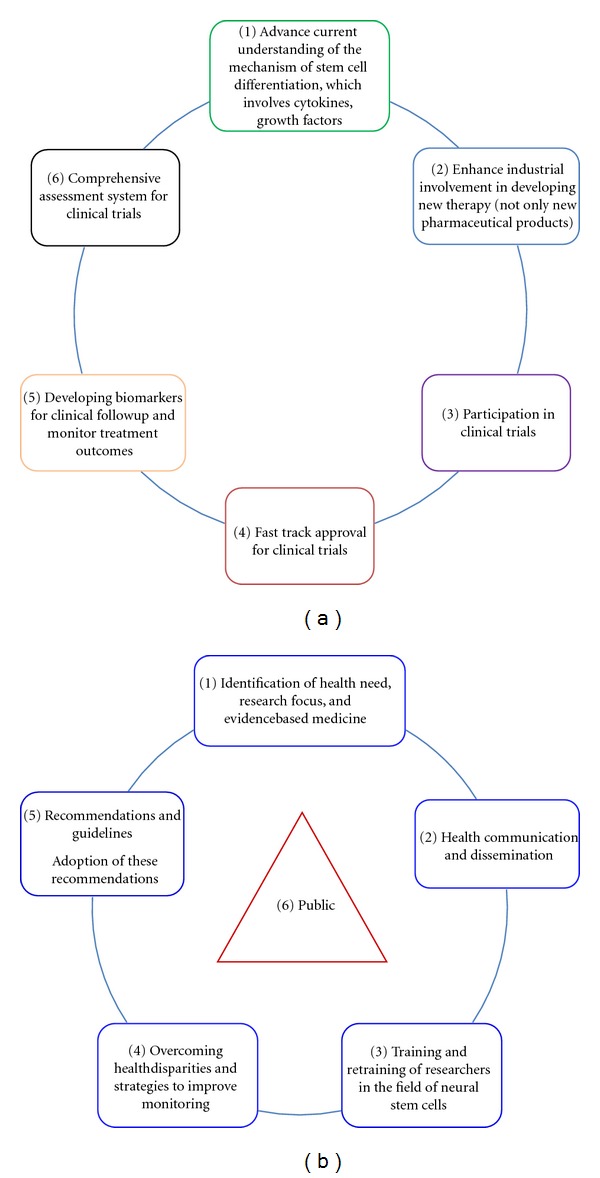
(a) Proposed schema for effective translation involving concerted effort of multilevel strategies of six main stakeholders. (b) Proposed framework for the reinforcement of the multi-level strategies effective bench to bedside translation of NSCs in TBI.

The authors, nonetheless, propose six-point schema for improving bench to bedside translation of stem cell therapy (Figure 3(a)) involving a rigorous network of six stakeholders: basic researchers, pharmaceutical companies, patient or general public participating in clinical trials, regulatory bodies or agencies for grant approval, collaborative research between basic and clinical scientist with the plan of developing biomarkers for potential drug targets, and creating a concerted network of groups that identifies some of the medical problems relating to TBI. We are still faced with the need to formulate hypothesis both at experimental and clinical epidemiologic levels and implementing these into clinical practice while the translational researcher serves to collaborate and coordinate all these strategies.

Indeed, communication and dissemination (Figure 3(b)) which are patient centeredness will not only impact on the public, but will also help to tame the ethical problems in this field. Communication will involve both patients and other clinicians involved in conducting randomized clinical trials (RCTs). With strong feedback on outcomes, pharmacovigilance, and health promotion, education of the populace in form of scientific advocacy is so paramount as this will impact on improved scientific collaboration, quality public control, and increased transparency among researchers and may improve funding of research work [69].

Research in neural stem cell is still a grey area and much knowledge needs to be gained, to actually close the gaps. There is inadequate understanding of secondary injury process, insufficient preclinical testing in diffuse axonal injury models, species differences, and lack of understanding of the mechanism of drug-receptor interactions. Smith and colleagues had suggested the need to use gyrencephalic models for proper translation of TBI [70]. There is need for increased linkages and networking between academician, researchers, and clinicians for greater reward of what is being generated.

Methodological disparities between experimental models of TBI and clinical studies cannot be overemphasized. The intent to treat models, differences in statistical analysis as a result of differences in sample size, and different behaviours between human and animals. Injury severities in animals differ from humans; while they are well defined in animals, they could take any direction in human. The need to improve study quality score has recently being called for by stroke therapy academic industry roundtable (STAIR), which was recently updated and this includes the following recommendations: (1) elimination of randomizations and assessment bias, (2) use of a priori definitions of inclusion/exclusion criteria, (3) inclusion of appropriate power and sample size calculations, (4) full disclosure of potential conflict of interest, (5) evaluation of therapies in male and female animals across the spectrum of ages, and with comorbid conditions such as hypertension and/or diabetes. Furthermore, some researchers had also expanded on these proposed recommendations for improved clinical trials in brain injury with special focus on neuroprotective therapies in TBI [70, 71]. Nonadherence was the single most important determinant of trial failure in the past.

Finally, the International Mission on Prognosis and Clinical Trial Design in TBI (IMPACT) proposed ways of overcoming the above disparities and challenges. The recommendations include a robust inclusion criteria and recommendations for general research in TBI [70]. The six-point schema is an overview recommendation with the public, patient, or the society as the core and the fulcrum of all activities of research and if implemented may yield quality research outcome in neural stem cells translation in TBI. 

## 5. Conclusions

Mortality and disability from TBI are projected to rise globally. Neural stem cell therapy is a strategy that offers hope in the future for treatment of brain injury. In addition, we are now able to monitor autologous neural stem cells *in vivo*, cell migration and clearly demonstrate that neural stem cells could selectively target injured brain or spinal cord tissue and undergo neurogenesis. Finally, the proposed six-points cyclical schema should be implemented with determined effort of all stakeholders for effective bench to bedside translation of neural stem cell therapy in TBI.
